# CCL20 and Beta-Defensin 2 Production by Human Lung Epithelial Cells and Macrophages in Response to *Brucella abortus* Infection

**DOI:** 10.1371/journal.pone.0140408

**Published:** 2015-10-08

**Authors:** M. Soledad Hielpos, Mariana C. Ferrero, Andrea G. Fernández, Josefina Bonetto, Guillermo H. Giambartolomei, Carlos A. Fossati, Pablo C. Baldi

**Affiliations:** 1 Instituto de Estudios de la Inmunidad Humoral (IDEHU, CONICET-UBA), Facultad de Farmacia y Bioquímica, Universidad de Buenos Aires, Buenos Aires, Argentina; 2 Instituto de Inmunología, Genética y Metabolismo (INIGEM, CONICET-UBA), Hospital de Clínicas "José de San Martín", Buenos Aires, Argentina; 3 Instituto de Estudios Inmunológicos y Fisiopatológicos (IIFP, CONICET-UNLP), Facultad de Ciencias Exactas, Universidad Nacional de La Plata, La Plata, Argentina; National University, COSTA RICA

## Abstract

Both CCL20 and human β-defensin 2 (hBD2) interact with the same membrane receptor and display chemotactic and antimicrobial activities. They are produced by airway epithelia in response to infectious agents and proinflammatory cytokines. Whereas *Brucella* spp. can infect humans through inhalation, their ability to induce CCL20 and hBD2 in lung cells is unknown. Here we show that *B*. *abortus* induces CCL20 expression in human alveolar (A549) or bronchial (Calu-6) epithelial cell lines, primary alveolar epithelial cells, primary human monocytes, monocyte-derived macrophages and the monocytic cell line THP-1. CCL20 expression was mainly mediated by JNK1/2 and NF-kB in both Calu-6 and THP-1 cells. CCL20 secretion was markedly induced in A549, Calu-6 and THP-1 cells by heat-killed *B*. *abortus* or a model *Brucella* lipoprotein (L-Omp19) but not by the *B*. *abortus* lipopolysaccharide (LPS). Accordingly, CCL20 production by *B*. *abortus*-infected cells was strongly TLR2-dependent. Whereas hBD2 expression was not induced by *B*. *abortus* infection, it was significantly induced in A549 cells by conditioned media from *B*. *abortus*-infected THP-1 monocytes (CMB). A similar inducing effect was observed on CCL20 secretion. Experiments using blocking agents revealed that IL-1β, but not TNF-α, was involved in the induction of hBD2 and CCL20 secretion by CMB. In the *in vitro* antimicrobial assay, the lethal dose (LD) 50 of CCL20 for *B*. *abortus* (>50 μg/ml) was markedly higher than that against *E*. *coli* (1.5 μg/ml) or a *B*. *abortus* mutant lacking the O polysaccharide in its LPS (8.7 ug/ml). hBD2 did not kill any of the *B*. *abortus* strains at the tested concentrations. These results show that human lung epithelial cells secrete CCL20 and hBD2 in response to *B*. *abortus* and/or to cytokines produced by infected monocytes. Whereas these molecules do not seem to exert antimicrobial activity against this pathogen, they could recruit immune cells to the infection site.

## Introduction

Airways epithelial cells and alveolar macrophages are the first cells contacted by inhaled microorganisms and are therefore prepared to mount rapid immune responses. Besides constituting an anatomical barrier for microbial invasion, the respiratory epithelium responds to the presence of pathogens with an inflammatory response, including cytokines and chemokines, aimed at controlling the infection [1, 2]. Such epithelial response may be further enhanced by the stimulating action of cytokines secreted by alveolar macrophages [3–5].

Factors produced by the respiratory epithelium in response to infections include beta-defensins, small antimicrobial peptides that can be found in the fluid lining the respiratory tract together with other antimicrobial components such as lysozyme and cathelicidins. Human beta-defensin 2 (hBD2) is the most highly expressed beta-defensin in the lung and its expression is up-regulated during infections or inflammation [6]. All defensins are small cationic, microbicidal peptides that contain six highly conserved cysteine residues which form three pairs of intramolecular disulfide bonds. It is postulated that these peptides are attracted by electrostatic forces to the negative charges on the membrane surface provided by lipopolysaccarides (LPS) in Gram-negative bacteria and by several components in Gram-positive bacteria. Then, they would interact with the lipid bilayer of the bacterial cytoplasmic membrane leading to alteration of the membrane structure and creation of a physical hole that causes cellular contents to leak out [7]. In particular, hBD2 has been shown to be effective in vitro against several pathogens, including *E*. *coli*, *P*. *aeruginosa*, *Klebsiella pneumoniae*, etc. [8].

The respiratory epithelium also responds to infections with the production of several chemokines, including CCL20. Alternatively named liver and activation-regulated chemokine (LARC), macrophage inflammatory protein-3 (MIP-3) or Exodus-1, CCL20 is a chemoattractant for immature dendritic cells, effector/ memory T-cells and B-cells. CCL20 and its specific receptor CCR6 have been shown to mediate *in vivo* the recruitment of dendritic cells and lymphocytes in several tissues, including the lung [9–11]. Of note, the repertoire of CCR6+ T cells recruited by CCL20 also includes Th17 cells [12], a fact that may be relevant for immune responses to infectious agents.

Notably, CCL20 and β-defensins, especially hBD2, have been found to share many similarities. Both factors have been shown to interact with the same membrane receptor, CCR6. While binding of CCL20 to this receptor was known to mediate the chemotactic responses of immature dendritic cells to this chemokine, more recent studies showed that β-defensins also display chemotactic activity by binding to CCR6 [13–16]. They can act as chemoattractants for several cells of the innate and adaptive immunity and can stimulate different immune responses (including cytokine secretion, dendritic cell maturation, etc.) [17–19]. In particular, hBD2 has been shown to induce the chemotaxis of memory T cells, immature dendritic cells, mast cells and neutrophils [15, 20, 21]. On the other hand, whereas CCL20 was initially described as a chemokine, more recent studies have revealed that this molecule can also display antimicrobial activities against Gram positive and Gram negative bacteria [22–24]. It has been postulated that the antimicrobial activity of CCL20 may be due to the fact that this chemokine shares structural properties with β–defensins, including antiparallel β–pleated sheet core structure and charge distribution [22].

The expression and/or production of CCL20 and hBD2 have been shown to increase in pulmonary epithelial cells in response to different infectious agents or antigens [25–31] and also in response to proinflammatory cytokines [22, 32–37].

Human brucellosis, mainly caused by *Brucella melitensis*, *B*. *suis* or *B*. *abortus*, is a worldwide distributed zoonotic disease which affects over 500,000 people annually [38, 39]. Due to the easy aerosolization of these bacteria, inhalation of infected aerosols is frequently involved in contagion. For this reason *Brucella* spp. are considered potential biological weapons [39] and have been classified by CDC and NIAID as category B bioterrorism agents. Airborne transmission has been implicated in outbreaks of human brucellosis in different settings [40, 41] and also in most cases of laboratory-acquired brucellosis [42, 43]. Despite the importance of the respiratory route for *Brucella* entry to the organism, the interaction of these bacteria with the pulmonary cells has been scarcely studied. We have previously shown that *Brucella* species can infect and replicate within human lung epithelial cells, and can induce them to produce the monocyte chemoattractant MCP-1 [44, 45].

Because of their chemotactic and antimicrobial activities, both CCL20 and beta-defensins are postulated to have important roles in the pulmonary innate immune response to inhaled pathogens [46–48], and several studies have shown the induction of CCL20 or hBD2 in lung tissues during infection. However, nothing is known about the expression of these molecules in *Brucella*-infected lung epithelial cells or about their antimicrobial activity against this pathogen. The purpose of the present study was to address these issues.

## Material and Methods

### Reagents

CCL20, hBD2 and hBD3 used in this study were recombinant proteins purchased from PeproTech (Rocky Hill, NJ). Lipopolysaccharide (LPS) from *Brucella abortus* 2308 was provided by Ignacio Moriyón (University of Navarra, Pamplona, Spain). The purity and the characteristics of this preparation have been published elsewhere [49]. The synthetic bacterial lipohexapeptide Pam3CSK4 was obtained from InvivoGen (San Diego, USA). Flagellin from *Salmonella enterica* serovar Typhimurium was provided by Martín Rumbo (Universidad de la Plata, Buenos Aires. Argentina). LPS from *Escherichia coli* O55:B5 was provided by Sigma (Taufkirchen, Germany). The lipidated 19 kDa outer membrane protein from *B*. *abortus* (L-Omp19) was obtained in recombinant form in *E*. *coli* as described previously [50]. Briefly, the open reading frame of Omp19 was cloned in the pET22b+ vector, and the resulting plasmid (pET-L-Omp19) containing the Omp19 gene with a COOH-terminal 6X histidine tag was used to transform competent *E*. *coli* BL21(DE3) bacteria. After induction with isopropyl β-D-thiogalactopyranoside, recombinant L-Omp19 was isolated from bacterial membranes by sonication and selective extraction with Triton X-114, and was further purified by affinity chromatography with a Ni-NTA resin. After adsorption with Sepharose-polymyxin B the recombinant protein contained less than 0.25 endotoxin U per μg of protein as assessed by Limulus Amebocyte Lysate Test (Associates of Cape Cod, East Falmouth, MA).

### Bacterial Strains and Growth Conditions

*B*. *abortus* 2308 and *B*. *abortus* RB51 (rough vaccine strain) were grown overnight in tryptic soy broth (TSB), harvested by centrifugation, and washed twice in phosphate-buffered saline (PBS). Bacterial numbers in cultures were estimated by comparing the OD at 600 nm with a standard curve, but the actual concentration of inocula was checked by plating on tryptic soy agar plates (TSA). To prepare heat-killed *B*. *abortus* (HKBA), bacteria were washed in sterile PBS, heat killed at 70°C for 30 min, aliquoted, and stored at -70°C until used. All live *Brucella* manipulations were performed in biosafety level 3 facilities.

### Cell Lines

The A549 cell line (human type II alveolar epithelial) and the human bronchial epithelial cell line Calu-6 were from the American Type Culture Collection (ATCC). A549 cells were grown in Dulbecco’s modified Eagle’s medium (DMEM) supplemented with 2 mM L-glutamine, 10% heat-inactivated FBS (Gibco, USA), 100 U/ml penicillin, 100 mg/ml streptomycin (supplemented DMEM). Calu-6 cells were grown in supplemented RPMI 1640 (Gibco). For infection assays, all epithelial cell lines were seeded at 2 x 10^5^ cells/well in 24 well plates and cultured in a 5% CO_2_ atmosphere at 37°C for 24 h in antibiotic-free culture medium.

### Human Lung Tissue Explants and Bacterial Infection

Pulmonary tissue samples of approximately 1 cm^3^ were obtained from healthy portions of human lungs resected during lung transplant. Tissue samples were kindly provided by Favaloro Hospital in Buenos Aires thanks to a Cooperation Agreement established with the authors’ institution (CONICET). Favaloro Hospital is the reference centre for lung and heart transplant in Argentina (www.fundacionfavaloro.org). The use of lung tissue samples was approved by the Institutional Review Boards of Favaloro Hospital (Departamento de Docencia e Investigación) and CONICET (Dirección de Vinculación Tecnológica) (study protocol N° 3099). As stated in the Agreement, written informed consent from patients was obtained by the surgeons. Samples were cultured in RPMI 1640 medium (Sigma) at 37°C and 5% CO_2_ and incubated with 500 μl *B*. *abortus* 2308 suspensions (10^7^ CFU/ml) or medium for 24 h.

### Isolation of Primary Cells from Lung Tissue

Type II alveolar epithelial cells (ATII cells) were isolated from lung tissue obtained as described above, following published procedures [51]. Briefly, the tissue was rinsed with Solution A (0.130 M NaCl, 5.2 mM KCl, 10.6 mM Hepes, 2.6 mM Na_2_HPO_4_, 10 mM D-glucose with pH adjusted to 7.4) to remove excess blood and debris. It was then perfused repeatedly with Solution A, using a syringe, until the perfusate was virtually free from leukocytes (10^4^ cell/ml perfusate). Lung tissue was minced into pieces of < 0.5 mm thickness. The tissue was digested with trypsin (0.5% in solution A plus 1.9 mM CaCl_2_ and 1.29 mM MgSO_4_) and incubated at 37°C for 20 min in a shaking water bath. The digest was filtrated through a 100 μm mesh and the filtrate was collected over fetal calf serum containing DNase (DNase I, Sigma; 400 U/ml). This suspension was filtered through a 40 μm mesh. The filtrate, containing single cells, was centrifuged and the type II cell–enriched pellet suspended in DMEM/SAGM containing 350 U/ml DNase, then plated into a large flask to allow adhesion of contaminating leukocytes and other cells. After 2 h, the non-adherent ATII cells were isolated from the medium supernatant by virtue of their buoyant density over a Percoll discontinuous gradient (1.089 g/l and 1.04 g/l; Sigma). The interfacial layer enriched in ATII cells was collected and washed twice with Solution A. Cells were stained with trypan blue and counted in a Neubauer chamber. For further experiments, a total of 2×10^5^ ATII cells/well were seeded in SAGM medium (Lonza, Wuppertal, Germany) onto 48 well plates coated with type I collagen. Cells were allowed to adhere for at least 36 h, then nonadherent cells were removed and media was changed every 24 h until confluence (4–5 d).

### Cellular Infections

*Brucella* infections of respiratory epithelial cells were performed at different multiplicities of infection (MOI). After dispensing the bacterial suspension the plates were incubated for 2 hours at 37°C under 5% CO_2_ atmosphere. At the end of the incubation time (time 0 p.i.), each well was washed three times with sterile PBS. To kill extracellular bacteria, the infected monolayers were incubated with 100 μg/ml of gentamicin (Sigma) and 50 ug/ml of streptomycin (Sigma). At different times after antibiotics addition culture supernatants were harvested to measure antimicrobial peptides by ELISA.

In experiments aimed at evaluating the role of TLR2 in cellular responses to infection, THP-1 and Calu-6 (0.5 x 10^6^ and 2.5 x 10^5^ cells/ml respectively) were incubated with 20 μg/ml of anti-human TLR2 (clone TL2.1; eBioscience, San Diego, CA) or an isotype-matched control for 30 min at 37°C before infection with *B*. *abortus* 2308. After this, culture supernatants were harvested as indicated above.

### Analysis of Antimicrobial Peptide Production in Response to Antigens

Respiratory epithelial cells (5 x 10^5^ cells/ml) cultured in 24-well plates were stimulated with different *B*. *abortus* antigens (HKBA, LPS, L-Omp19) or with TLR agonists (*E*. *coli* LPS, Pam3CSK4). Cultures were incubated for 24 h. At the end of the culture, supernatants were harvested, aliquoted and stored at -70°C until they were analyzed for antimicrobial peptides.

### Stimulation with Conditioned Media

THP-1 monocytes were infected with *B*. *abortus* 2308 at an MOI of 100 for 2 h, and then washed with sterile PBS to remove extracellular bacteria. Conditioned media from infected monocytes (CMB) were harvested at 24 h p.i., sterilized by filtration through 0.22 μm nitrocellulose filters, and used to stimulate epithelial cells. Conditioned media from uninfected human monocytes (CMC) cultured for 24 h served as negative controls. For stimulation, conditioned media were used diluted 1/2, 1/5, or 1/10 in complete medium. At 24 h of stimulation, the supernatants from stimulated cultures were harvested to measure antimicrobial peptides. To calculate the specific secretion of each antimicrobial peptide, levels already present in the transferred conditioned media were subtracted from levels measured after stimulation. Neutralization experiments were performed with the IL-1 receptor antagonist (IL-1Ra, provided by R&D Systems, Minneapolis, MN) or an anti-TNF-α neutralizing antibody (BD Biosciences, San Diego, CA). Epithelial cells were preincubated for 1 h with IL-1Ra before the addition of conditioned media, or the CMB was preincubated with anti-TNF-α neutralizing antibody for 1 h before being added to epithelial cells.

### Semiquantitative Reverse Transcriptase/Polymerase Chain Reaction

Transcription levels of mRNA for hBD2 were determined by semiquantitative RT-PCR using previously described primers [52]. Epithelial cells were washed twice with phosphate-buffered saline and total RNA was isolated using the TRIzol reagent (Invitrogen, Life Technologies). Total RNA (2 μg) was reverse transcribed using standard reagents (Invitrogen). The complementary DNA (cDNA) corresponding to 200 ng RNA was amplified in a polymerase chain reaction (PCR) containing 0.1 mM of hBD-2 specific intron spanning primers (forward primer: 5´-CCAGCCATCAGCCATGAGGGT-3´; reverse primer: 5´-GGAGGCCTTTCTGAATCCGCA-3´, product: 255 bp) with an internal control for equal amounts of cDNA of a glyceraldehyde- 3-phosphate dehydrogenase (GAPDH)–specific intron spanning primer pair (5´-CCAGCCGAGCCACATCGCTC-3´; 5´-ATGAGCCCCAGCCTTCTCCAT-3´), which yielded a 360- bp amplified product. Amplification was performed using 25 to 40 cycles with denaturation at 94°C for 30s, primer annealing at 60°C for 30s, and extension at 72°C for 1 min. PCR products were subjected to electrophoresis on a 2% agarose gel and visualized by SYBR Safe staining (Invitrogen). Specificity of hBD-2 encoding PCR products was verified by sequencing.

### Measurement of Antimicrobial Peptides

Human CCL20 and hBD2 were measured in culture supernatants of cells or lung tissue explants infected with *Brucella* or stimulated with *Brucella* antigens by sandwich ELISA (R&D, Minneapolis or Peprotech respectively) using paired cytokine-specific mAbs, according to the manufacturer’s instructions.

### Signaling Pathways in CCL20 Production

To examine the signaling pathways involved in CCL20 secretion, THP-1 and Calu-6 cells were pretreated with SB203580 (p38 MAPK inhibitor), PD98059 (Erk1/2 MAPK inhibitor), SP600125 (Jnk1/2 inhibitor) BAY 11-7082 (NF-κB inhibitor) or vehicle (dimethyl sulfoxide, DMSO). The inhibitors were added one hour before infection with *B*. *abortus* and were kept throughout the experiment (24 h). Cell viability after incubation with these inhibitors was higher than 90%, as assessed by staining with trypan blue. To account for any possible effect of DMSO on cell viability, cell cultures not treated with the inhibitors were treated with the highest final concentration of DMSO used in these studies (0.01%), and the results were compared with those of cell cultures not exposed to DMSO.

### Antimicrobial Assay

A standard colony-forming assay (CFA) was used [23], with slight modifications. Briefly, approximately 10^4^ CFU log phase microorganisms were mixed with CCL20, hBD2 or hBD3 at different final concentrations (0.1 to 50 μg/ml) in 0.1 ml of 10 mM potassium phosphate buffer (pH 7.4) containing 1% TSB. For the antibacterial assay, the mixture was incubated for 2 h at 37°C in shaking incubator (250 rpm). After serial dilution with 10 mM potassium phosphate buffer (pH 7.4) containing 1% TSB, the diluted mixture was plated in triplicate on TSA. The plates were incubated for 72 h at 37°C, and the colonies formed were counted.

## Results

### *B*. *abortus* Strain Induces CCL20 but Not hBD2 Secretion in Airway Epithelial Cells and Macrophages

**Fig 1:**
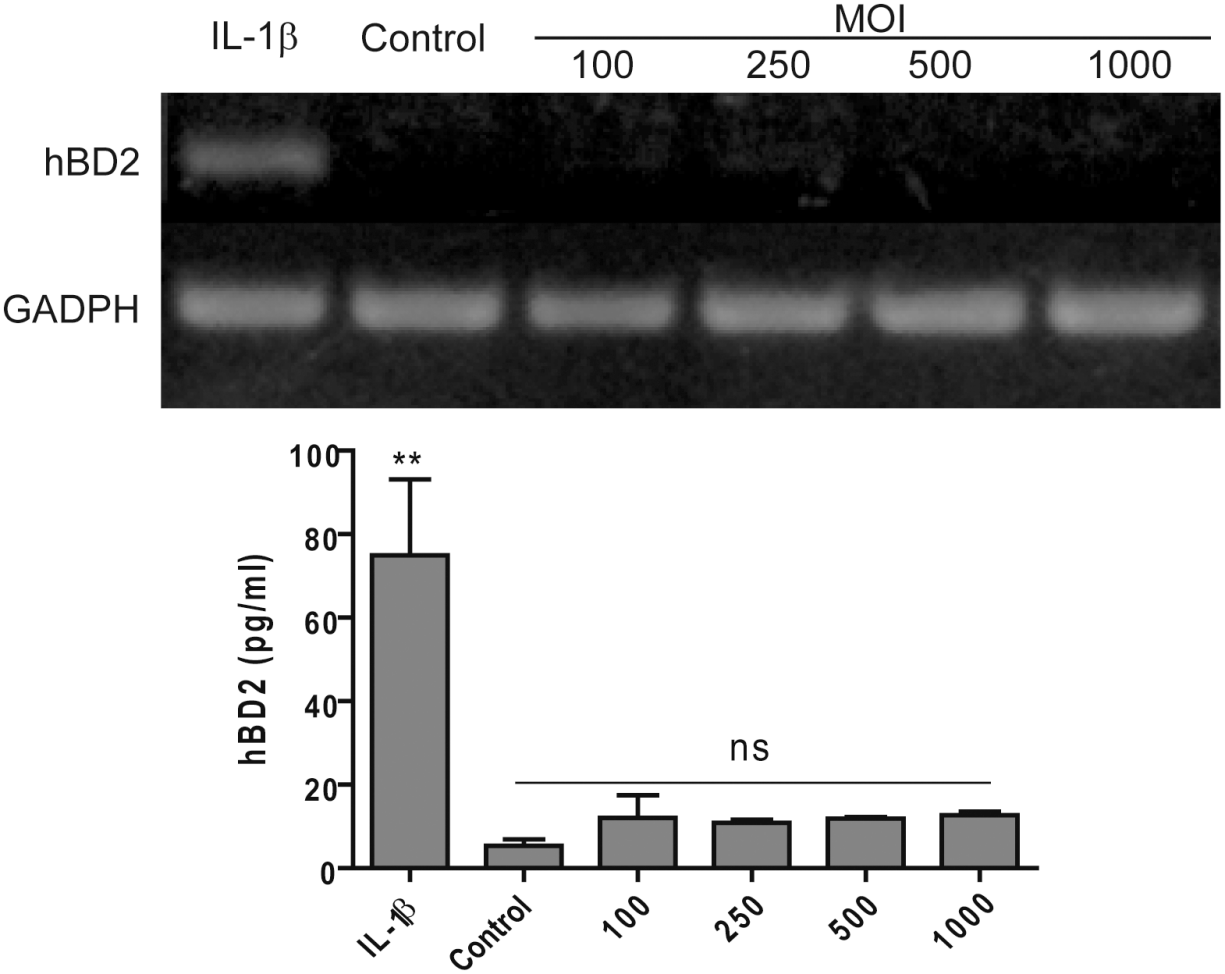
Human alveolar epithelial cells do not produce hBD2 in response to *B*. *abortus* infection. A549 cells were infected with *B*. *abortus* at different multiplicities of infection (MOI). At 48 h p.i. transcription levels of mRNA for hBD2 were determined by semiquantitative RT-PCR (upper panel), and culture supernatants were harvested for hBD2 determination by commercial ELISA (lower panel). As a positive control of hBD2 induction, cells were stimulated in parallel with recombinant IL-1β. ELISA results are the mean ± SEM of three independent experiments. **, p<0.01 and ns, non-significant difference versus untreated (control) cells (ANOVA followed by Dunnett’s test).

As airway epithelial cells have been shown to increase the expression of hBD2 and/or CCL20 in response to infection with several pathogens [28, 29, 34, 53, 54], we sought to determine whether infection with virulent *B*. *abortus* 2308 also increases the expression of hBD2 and CCL20 in these cells. Analysis by RT-PCR and ELISA revealed that *B*. *abortus* 2308 did not induce mRNA expression or protein secretion of hBD2 on the alveolar cell line A549 (Fig 1) or the bronchial cell line Calu-6 (data not shown), although both cell lines were able to express hBD2 mRNA in response to proinflammatory cytokine IL-1β (positive control).

**Fig 2:**
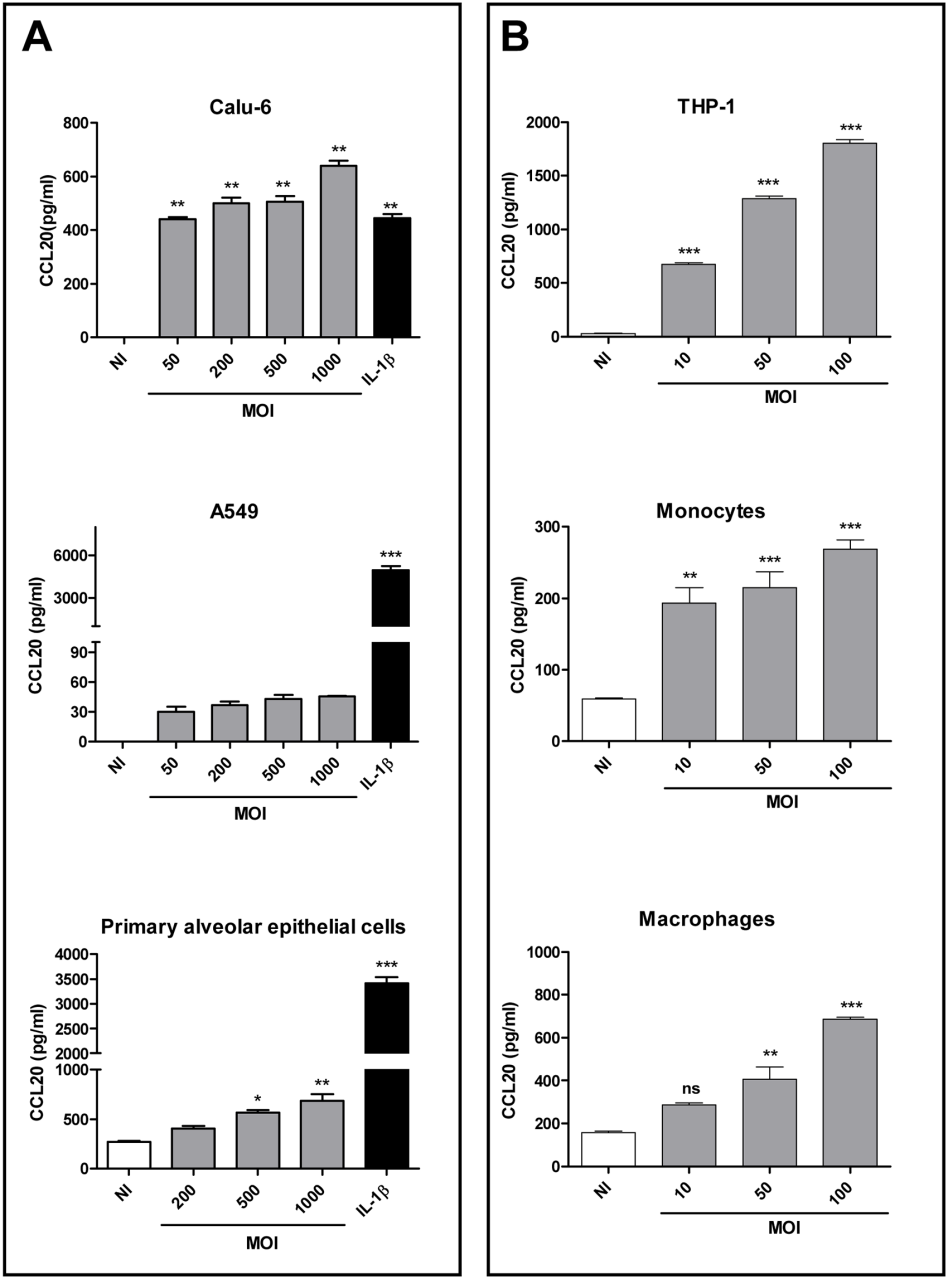
*B*. *abortus* induces CCL20 secretion by airways epithelial cells and human monocytes. (A) Calu-6 and A549 cell lines and primary alveolar epithelial cells were infected with *B*. *abortus* at MOIs 50 to 1,000 or were exposed to IL-1 β (1ng/ml) or media alone. (B) THP-1 cells, human peripheral blood-derived monocytes and monocyte derived-macrophages were infected with *B*. *abortus* at MOIs 10 to 100 or left untreated. Cell culture supernatants were harvested at 48 h, and CCL20 levels were measured by ELISA. Results are the mean ± SEM of three independent experiments. *, p<0.05; **, p<0.01; ***, p<0.001; and ns, non-significant difference versus non-infected cells (NI) (ANOVA followed by Dunnett’s test).

In contrast, infection with *B*. *abortus* increased CCL20 secretion in a MOI dependent manner in both Calu-6 cells and A549 cells, although chemokine levels were much lower in the later case (Fig 2A, upper and middle panels). CCL20 levels produced by primary cultures of human alveolar epithelial cells in response to *B*. *abortus* infection were similar to those produced by the Calu-6 bronchial cell line (Fig 2A, bottom panel).

The production of hBD2 and CCL20 in response to *B*. *abortus* infection was also evaluated in cells of the monocyte/macrophage lineage. Infection of the monocytic cell line THP-1 with *B*. *abortus* 2308 at MOI 1:10 and 1:100 did not induce hBD2 mRNA expression at any time point (data not shown). In contrast, *B*. *abortus* infection induced a significant increase of CCL20 secretion by THP-1 cells in a MOI dependent manner (Fig 2B, upper panel). In line with these results, primary culture blood monocytes and monocyte-derived macrophages produced significant amounts of CCL20 in response to *B*. *abortus* infection (Fig 2B, middle and bottom panels).

**Fig 3:**
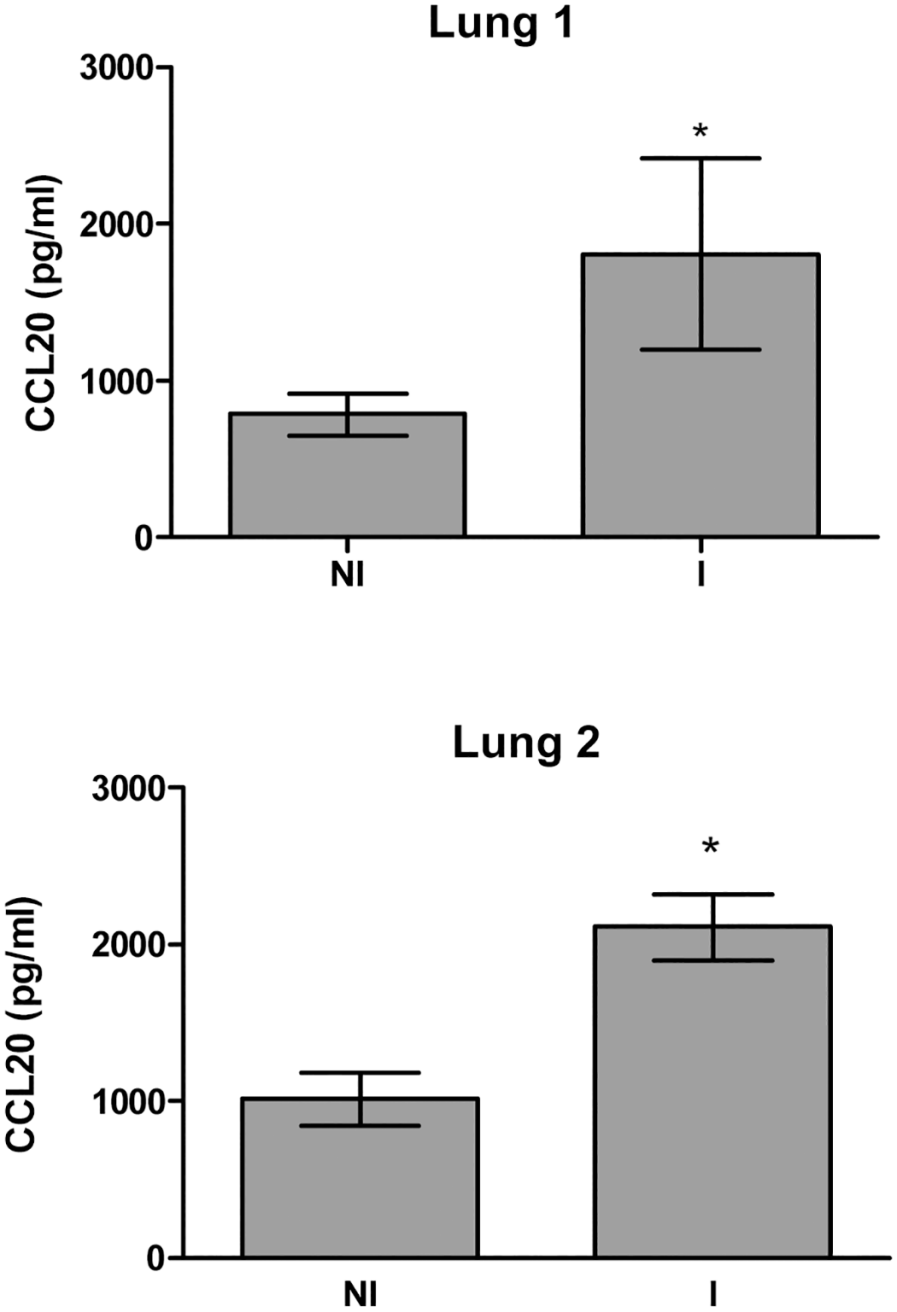
*B*. *abortus* induces CCL20 secretion in human lung tissue explants. Samples of healthy lung tissue (approximately 1 cm^3^) from two donors (Lung 1 and Lung 2, respectively) were incubated with a *B*. *abortus* 2308 suspension (10^7^ CFU/ml) or medium for 24 h. CCL20 levels were determined by ELISA in culture supernatants from infected (I) and non-infected (NI) explants. Results are the mean ± SEM of triplicate determinations for each sample. *,*P* < 0.05 as compared with the non-infected explant from the same donor (Student *t* test).

To further address the physiological relevance of CCL20 secretion by lung cells, the secretion of this chemokine by *Brucella*-infected human lung tissue explants was also assessed. As shown in Fig 3, after 24 h of growth in serum- free media, the levels of CCL20 in culture supernatants of infected explants were significantly higher than those produced by non-infected control explants (*P* <0.05).

### JNK and NF-κB Signaling Pathways Are Involved in the Secretion of CCL20 by *Brucella* Infected Lung Epithelial Cells and Macrophages

**Fig 4:**
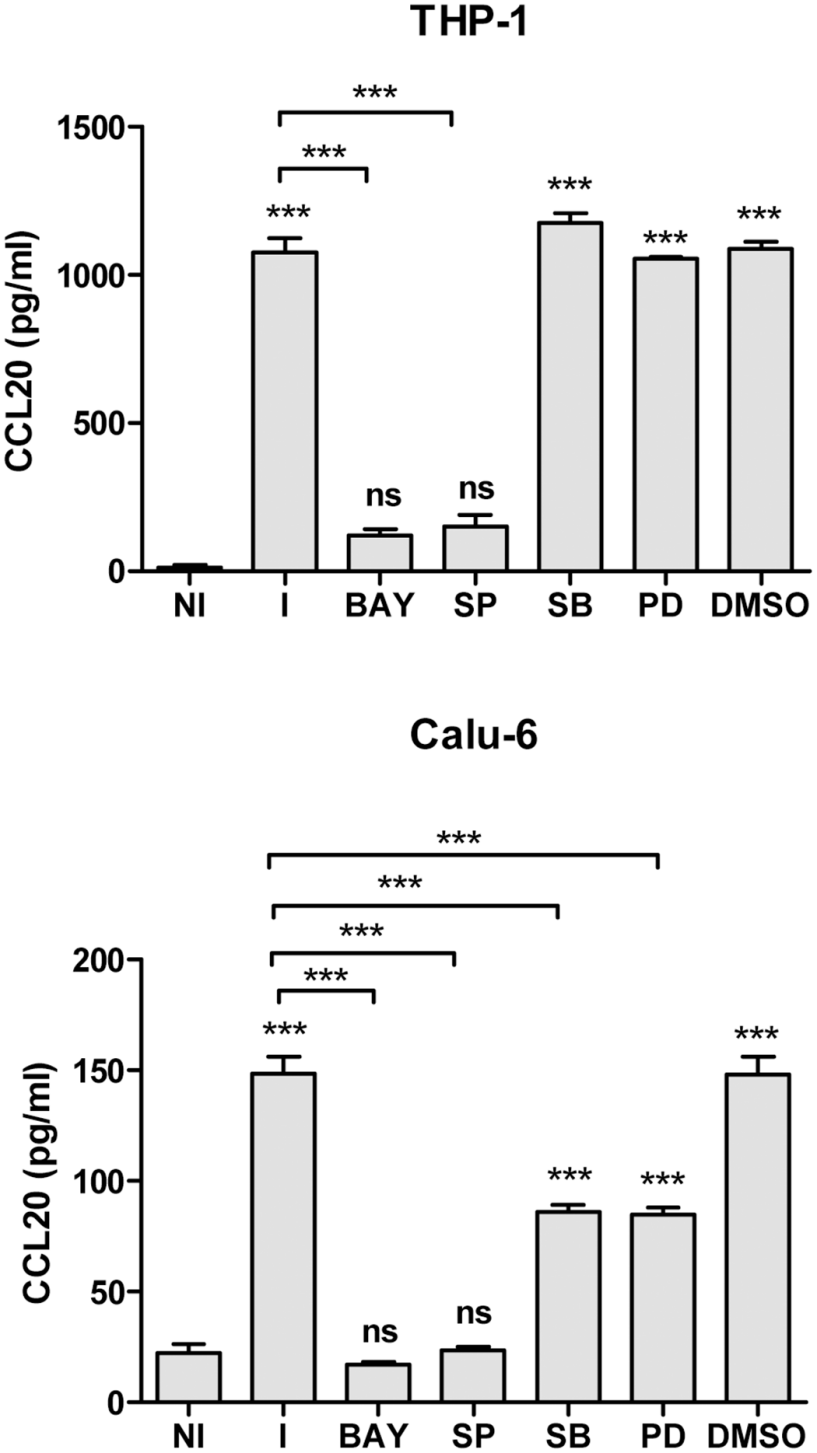
Role of MAPK pathways and NF-κB in CCL20 secretion by *Brucella*- infected lung epithelial cells and monocytes. Calu-6 and THP-1 cells were incubated with inhibitors of MAPK pathways (PD: inhibitor of Erk1/2; SB: inhibitor of p38 and SP: inhibitor of Jnk1/2) or NF-κB pathways (BAY) 1 h before the beginning of infection and kept throughout. Culture supernatants were harvested at 24 h post infection. NI: non-infected cells. Data are mean ± SEM of four independent experiments. Asterisks above bars indicate statistically significant differences (***, p < 0.001) relative to NI (ANOVA followed by Tukey’s Multiple Comparison test).

MAPK and NF-κB signaling pathways have been shown to be involved in the CCL20 response to infections or stimulation with microbial antigens, but the contribution of each pathway varies according to the cell type and/or the pathogen considered [30, 55]. To determine the potential role of p38, JNK1/2, ERK1/2, and NF-κB signaling pathways in CCL20 production by lung epithelial cells and macrophages in response to *B*. *abortus* infection, inhibition experiments were performed with the specific inhibitors SB203580, SP600125, PD98059 and BAY 11-7082, respectively. The production of CCL20 was strongly reduced (*P* < 0.001 as compared to untreated infected cells) by the JNK1/2 inhibitor and the NF-κB inhibitor in both Calu-6 and THP-1 cells, so that CCL20 levels produced by infected cells pretreated with these inhibitors did not differ significantly from those produced by uninfected controls (Fig 4). Conversely, inhibition of p38 and ERK1/2 had no effect on CCL20 secretion by *Brucella* infected-THP-1 monocytes but produced a partial inhibition in *Brucella-*infected Calu-6 cells. These results suggest that JNK1/2 and NF-κB pathways are involved in CCL20 responses to *B*. *abortus* infection in both cell types, whereas p38 and ERK1/2 pathways may also have a role in the response of Calu-6 cells.

### *B*. *abortus*-Induced Secretion of CCL20 Is Mediated by L-Omp19, but Not by *B*. *abortus* LPS

**Fig 5:**
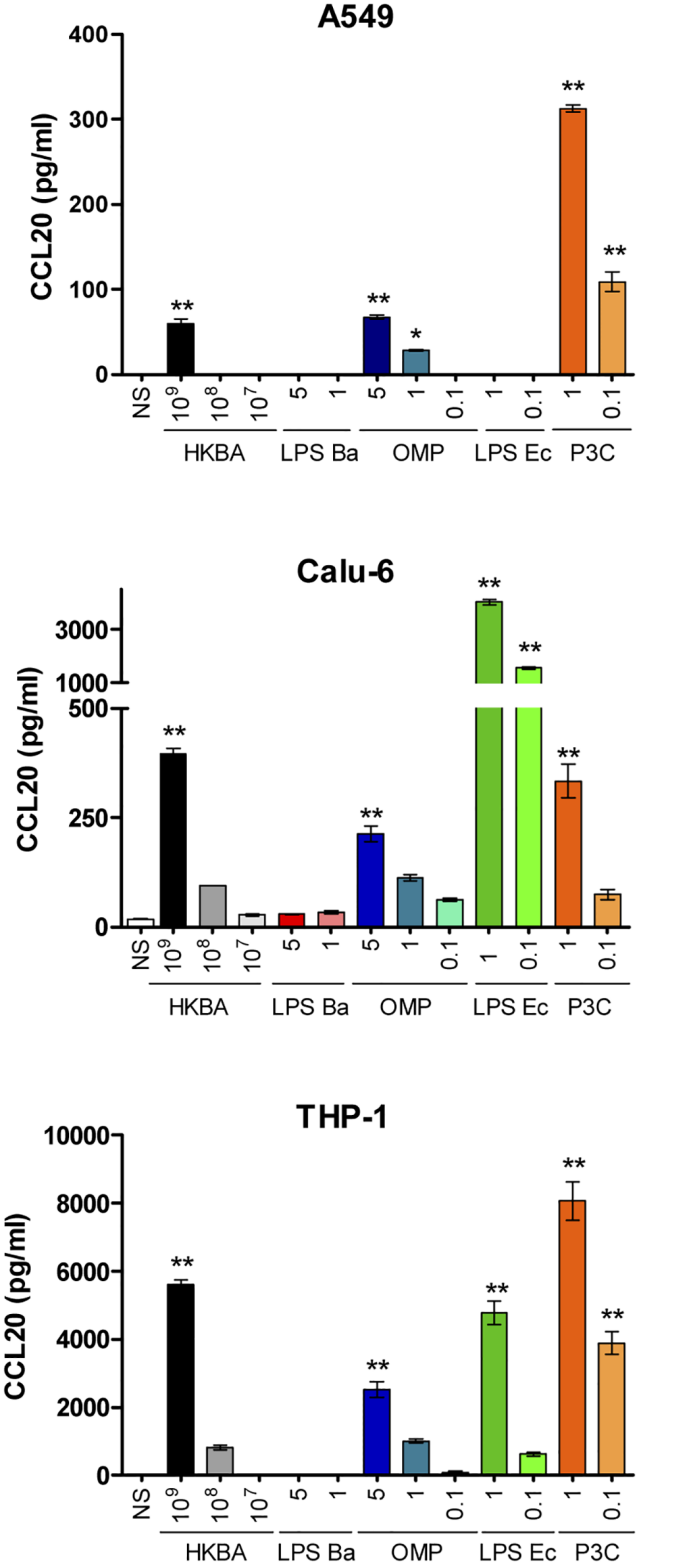
CCL20 production by lung epithelial cells and monocytes in response to *B*. *abortus* antigens and TLR agonists. Cells were stimulated for 24 h with heat-killed *B*. *abortus* (HKBA), the L-Omp19 lipoprotein (OMP, recombinant form obtained in *E*. *coli*) or the LPS from *B*. *abortus* (LPS Ba), or with the TLR agonists LPS from *Escherichia coli* (LPS Ec), and the lipohexapeptide Pam3CSK4 (P3C) (agonists for TLR4 and TLR2 respectively). The concentration of CCL20 in culture supernatants was determined by ELISA. Data are expressed as mean ± SEM of values from three independent experiments. Asterisks indicate statistical significant differences (*, p < 0.05; **, p < 0.01) as compared to non-stimulated cells (NS). (ANOVA followed by Dunnett’s Multiple Comparison test).

To test whether *B*. *abortus* viability is necessary to induce CCL20 production by lung epithelial cells and macrophages, the ability of heat-killed *B*. *abortus* (HKBA) to induce CCL20 was examined. As LPS from *Escherichia coli* is known to induce CCL20 expression in different cell types, including type II pneumocytes [31, 55] it was used as a positive control. The secretion of CCL20 was markedly enhanced in culture supernatants from A549, Calu-6 and THP-1 cells stimulated with HKBA when compared with unstimulated cells (Fig 5). For all cell lines a significant (*P* < 0.01) CCL20 secretion was detected in cultures stimulated with 1×10^9^ bacteria/ml. These results suggest that the secretion of CCL20 can be induced by a structural component of *B*. *abortus*. As it has been seen demonstrated that *B*. *abortus* lipoproteins induce cytokine production in different cells types [50, 56], we hypothesized that lipoproteins could also mediate CCL20 production. To investigate this hypothesis we used recombinant L-Omp19, a model *Brucella* lipoprotein. A549, Calu-6 and THP-1 cells were incubated with L-Omp19 obtained in recombinant form in *E*. *coli*, and culture supernatants were harvested 24 hours later to measure the secretion of CCL20. As shown in Fig 5, L-Omp19 induced a significant (*P* < 0.01) secretion of CCL20 in a dose-dependent fashion in all the three cell types under study. The production of CCL20 was also induced in all cells upon incubation with a synthetic lipohexapeptide (Pam3SCK4) that mimics the structure of the lipid moiety of bacterial lipoproteins. In contrast, *B*. *abortus* LPS did not induce CCL20 production even when used at high doses (5,000 ng/ml). Altogether, these results indicate that *B*. *abortus* lipoproteins are involved in the induction of CCL20 secretion by lung epithelial cells and monocytes in contact with this bacterium.

### TLR2 Recognition Mediates CCL20 Secretion by *Brucella*-Infected Cells

**Fig 6:**
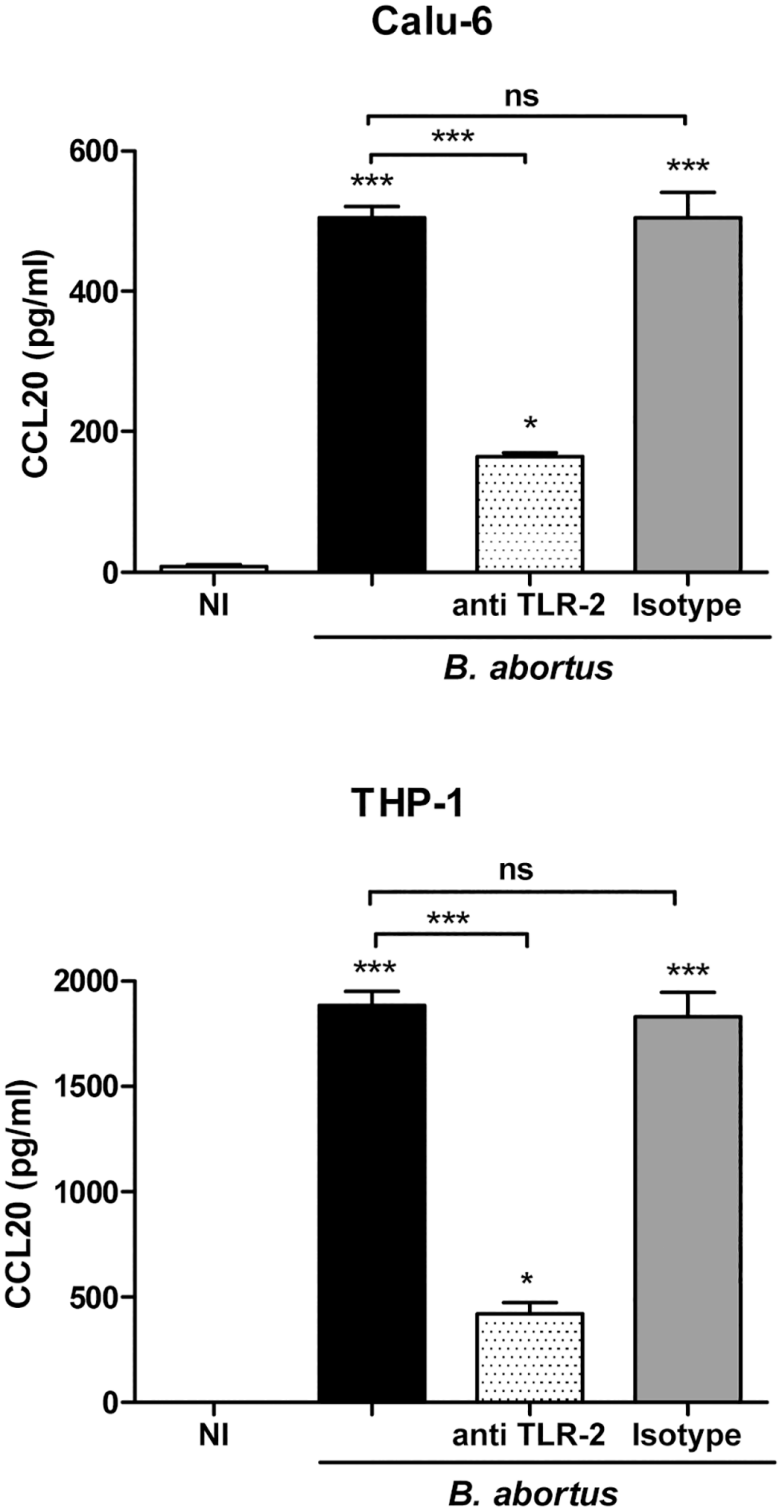
TLR2 recognition mediates CCL20 secretion by *B*. *abortus*-infected bronchial epithelial cells and monocytes. Calu-6 and THP-1 cells were preincubated with a neutralizing anti-TLR2 antibody or an isotype control for 1 h at 37°C before infection with *B*. *abortus*. Culture supernatants were harvested at 24 h p.i. to measure CCL20 by ELISA. Data are mean ± SEM of CCL20 levels measured in three independent experiments. Asterisks indicate statistical significant differences (*, p < 0.05; ***, p < 0.001) as compared to non-infected cells (NI) (ANOVA followed by Tukey’s Multiple Comparison test).

As our results indicate that *Brucella* lipoproteins, but not its LPS, induce CCL20 secretion by lung epithelial cells and monocytes, we hypothesized that TLR2 recognition may be involved in the signaling network leading to the secretion of this chemokine in *B*. *abortus*-infected cells. To test this hypothesis, THP-1 monocytes and Calu-6 epithelial cells were incubated with a blocking anti-TLR2 antibody before infection with live *B*. *abortus* 2308 (Fig 6). Blocking of TLR2 significantly reduced the CCL20 secretion of the infected cells (67.5% for Calu-6 and 77.9% for THP-1 cells), while incubation with an isotype control did not modify the CCL20 secretion in any cell type.

### Factors Secreted by *B*. *abortus*-Infected Monocytes Enhance the Secretion of CCL20 and hBD2 by Alveolar Epithelial Cells

It has been shown that airways epithelial cells release CCL20 and HBD2 in response to TNF-α and IL-1β [34, 57]. In the lung, resident alveolar macrophages and monocytes recruited to the infectious focus may respond to *B*. *abortus* with proinflammatory cytokines that, in turn, could stimulate the CCL20 and hBD2 production by airways epithelial cells. To test this hypothesis, A549 cells were stimulated with conditioned media from *B*. *abortus*-infected THP-1 monocytes (CMB) used at different dilutions. After 24 h of stimulation CCL20 and hBD2 were measured in culture supernatants by ELISA. Other A549 cells were stimulated in parallel with conditioned medium from uninfected monocytes (CMC) at the same dilutions. Levels of CCL20 (2150 ± 60.2 pg/ml) and hBD2 (14.4 ± 1.0 pg/ml) already present in the CMB were subtracted to determine specific stimulation (levels of both molecules were undetectable in CMC).

**Fig 7:**
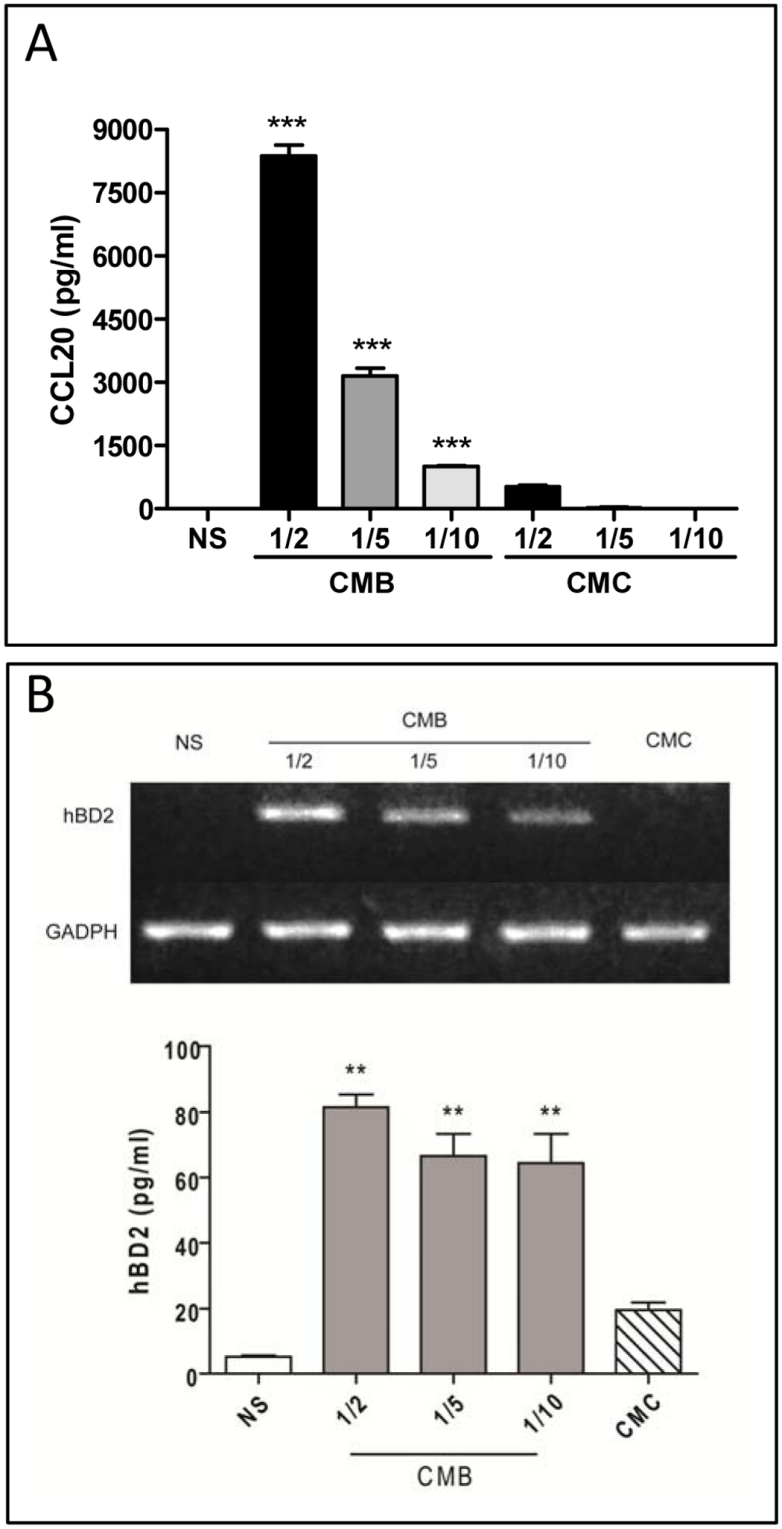
Factors secreted by *B*. *abortus*-infected monocytes enhance the secretion of CCL20 and hBD2 by alveolar epithelial cells. (A) Cells were exposed to different dilutions of conditioned medium from *B*. *abortus*-infected THP-1 monocytes (CMB) or from uninfected monocytes (CMC), and 24 h later the levels of CCL20 were measured in culture supernatants. (B) Cells were treated as above and 24 h later transcription levels of mRNA for hBD2 were determined by semiquantitative RT-PCR (upper panel), and culture supernatants were harvested for measuring hBD2 levels by commercial ELISA (lower panel). ELISA data are mean ± SEM of CCL20 or hBD2 levels measured in three independent experiments in stimulated cultures after subtracting preexisting levels in the transferred CMB or CMC. **, p<0.01, ***, p<0.001, and ns, non-significant difference versus non-stimulated (NS) cells (ANOVA followed by Dunnett’s test).

Whereas as shown above *B*. *abortus* infection did not induce a significant increase of hBD2 secretion in A549 alveolar epithelial cells and induced only low levels of CCL20, CMB stimulation resulted in a significant increment in the levels of both substances in culture supernatants from A549 cells. Mean levels of CCL20 were 8,377 ± 357.8 pg/ml in cells stimulated with CMB at 1/2, whereas they reached only 524 ± 50 pg/ml in cells stimulated with CMC and no level was detected under unstimulated conditions (Fig 7A).

A similar stimulatory effect was observed for hBD2. No mRNA expression was detected in A549 cells under unstimulated conditions, but CMB stimulation at different dilutions induced a marked expression of hBD2 mRNA (no induction was observed upon stimulation with CMC) (Fig 7B). To correlate hBD2 mRNA expression with protein abundance, levels of hBD2 were determined by ELISA in culture supernatants from CMB- or CMC-stimulated A549 cells. As shown in Fig 7B, the addition of CMC did not stimulate hBD2 secretion as compared to basal values, whereas CMB added at 1/2, 1/5 or 1/10 dilution increased hBD2 secretion by 81.5 ± 5.5, 66.7 ± 9.4 and 64.5 ± 12.5 pg/ml, respectively. These data indicate that CCL20 and hBD2 secretion from CMB-stimulated alveolar epithelial cells is much greater than that following direct infection of cells with *B*. *abortus*.

**Fig 8:**
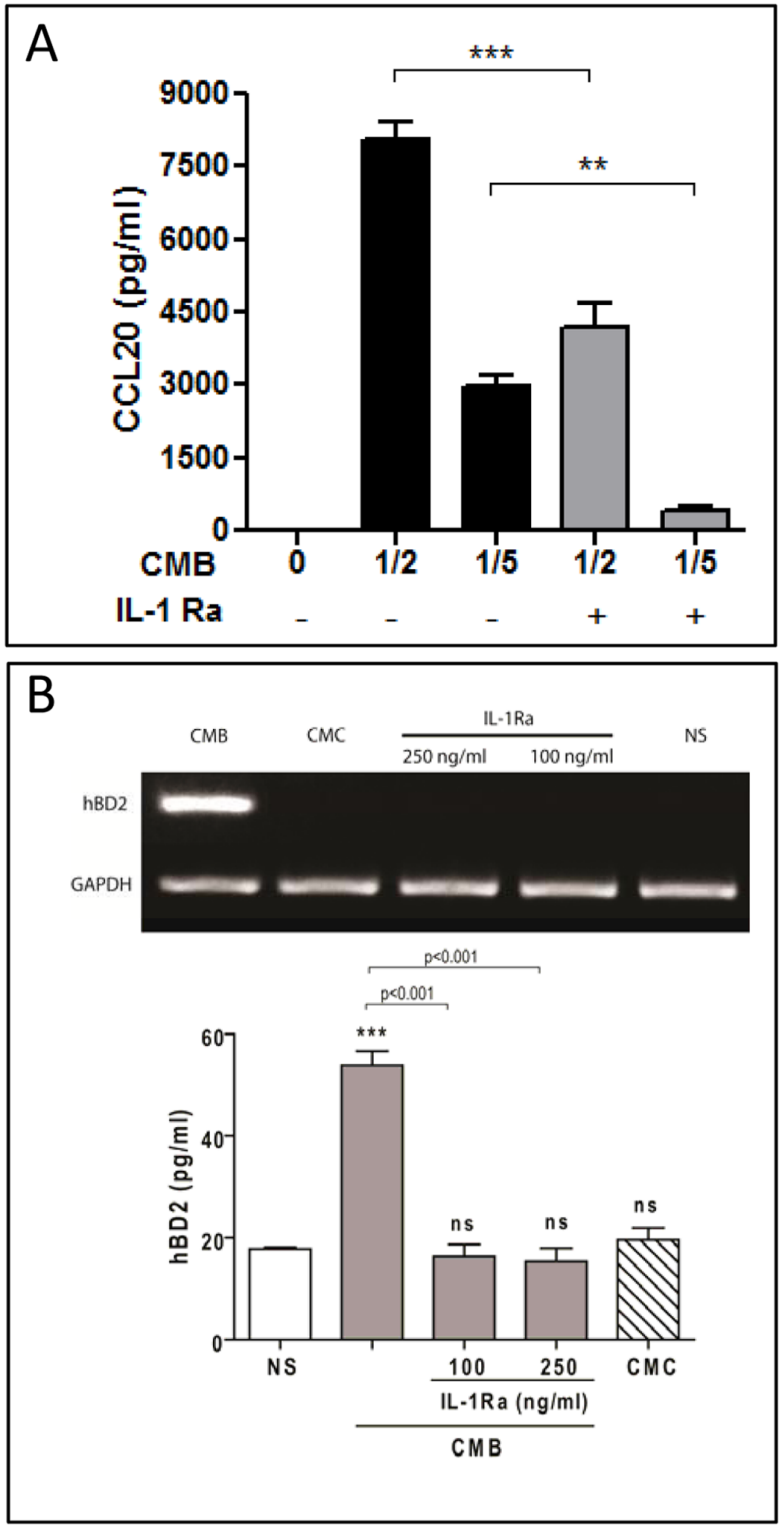
IL-1β mediates the stimulating effect of CMB on CCL20 and hBD2 secretion by alveolar epithelial cells. A549 cells were preincubated with IL-1Ra for 1 h at 37°C before stimulation with conditioned medium from *B*. *abortus*-infected THP-1 monocytes (CMB) or from uninfected monocytes (CMC) (at 1/2 and 1/5 dilution in A, at 1/5 dilution in B). At 24 h post-stimulation CCL20 levels (A) and hBD2 levels (B) were measured in culture supernatants, and the expression of mRNA for hBD2 was determined in cells. ELISA data are mean ± SEM of CCL20 or hBD2 levels measured in three independent experiments. **, p<0.01, ***, p<0.001, and ns, non-significant difference versus non-stimulated (NS) cells (ANOVA followed by Dunnett’s test).

As both TNF-α and IL-1β are known to induce CCL20 and hBD2 secretion by pulmonary epithelial cells [34, 57, 58] and are released by *B*. *abortus-*infected monocytes and alveolar macrophages [45, 59], they were considered potential candidates for mediators of the effects of CMB on alveolar epithelial cells. A neutralizing anti-human TNF-α antibody and IL-1Ra, the naturally occurring competitive inhibitor of IL-1β bioactivity, were used to test this hypothesis. Pretreatment of CMB with anti-TNF-α antibody did not reduce the CCL20 or hBD2 secretion of CMB-stimulated A549 cells (data not shown). However, treatment of A549 cells with IL-1Ra before stimulation with CMB at 1/2 or 1/5 dilution reduced CCL20 secretion by 48.3% and 86.3%, respectively (Fig 8A). Similarly, hBD2 secretion by cells stimulated with CMB at 1/5 was reduced by 63.6% after IL-1Ra treatment, whereas mRNA transcription levels were reduced to non-detectable (Fig 8B). These results suggest that IL-1β is essential for CCL20 and hBD2 production by CMB-stimulated A549 cells.

### Antimicrobial Activity

**Fig 9:**
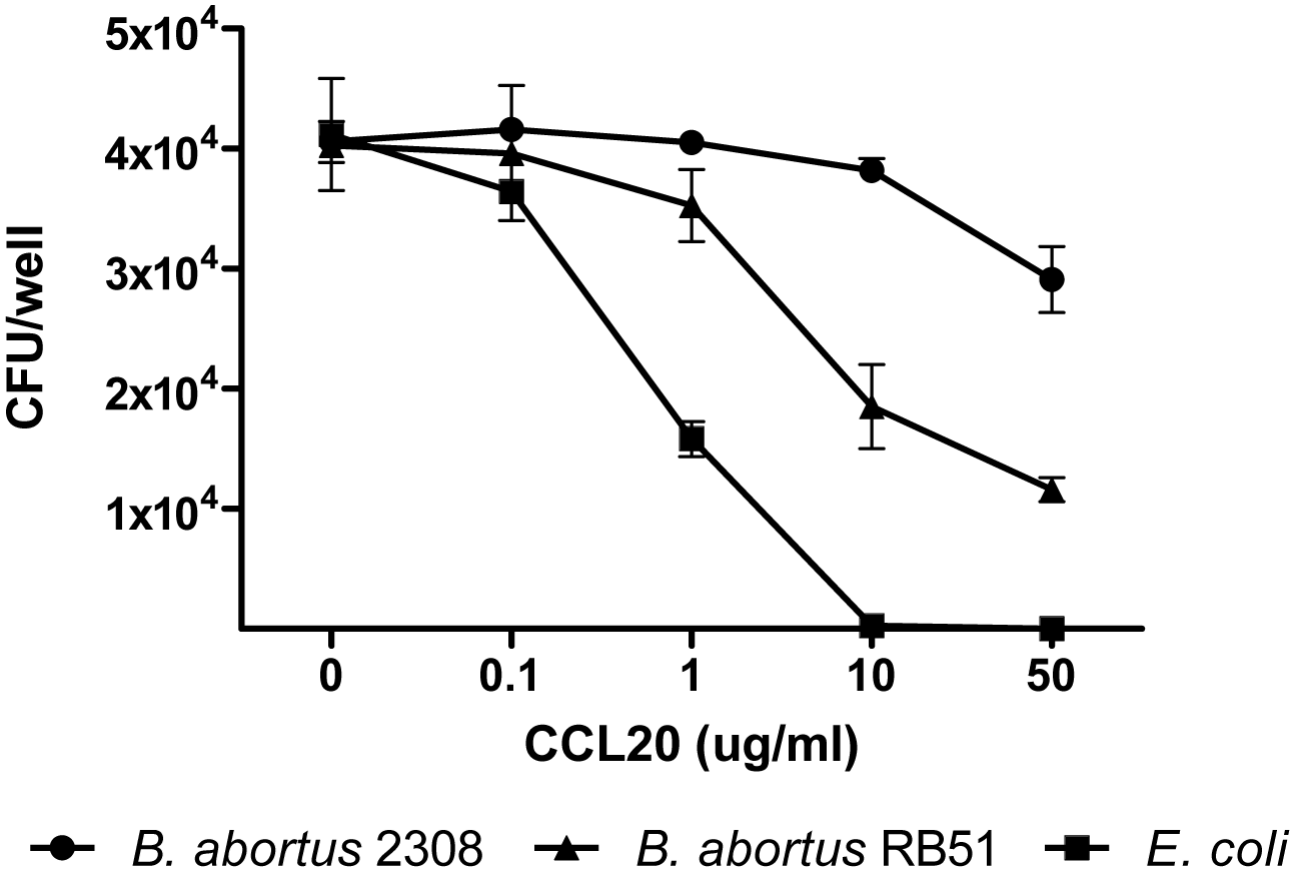
Antimicrobial activity of CCL20. *B*. *abortus* 2308, *B*. *abortus* RB51, and *E*. *coli* 25922 were incubated with various concentrations of recombinant CCL20 for 2 h and CFU were determined by plating serial dilutions of the mixture on agar. Data shown are means ± SEM of CFU counts from three independent experiments.

CCL20 and hBD2 were tested for antimicrobial activity against *B*. *abortus* 2308 using the standard colony-forming assay after coincubation of 10^5^ CFU bacteria in 0.1 ml 10 mM potassium phosphate buffer (pH 7.4) with various concentrations of human CCL20 and hBD2. CCL20 killed *B*. *abortus* 2308 with a lethal dose (LD) 50 (the dose that achieves 50% reduction of CFU) > 50 ug/ml (Fig 9). The capacity of CCL20 to kill *E*. *coli* 25922 was also examined as a positive control. CCL20 demonstrated anti-*E*. *coli* activity with a LD50 of 1.5 μg/ml. Given the comparative resistance of *B*. *abortus* to CCL20 lysis, we asked whether the presence of O polysaccharide in its LPS may exert a protective effect. To test this we evaluated the antimicrobial activity of CCL20 against *B*. *abortus* RB51, a rough vaccine strain devoid of O polysaccharide. As seen in Fig 9, CCL20 killed *B*. *abortus* RB51 in a dose-dependent manner with a LD50 of 8.7 ug/ml, suggesting that the O polysaccharide is involved in the relative resistance of *B*. *abortus* to the antimicrobial effects of CCL20. In parallel experiments, hBD2 and hBD3 (data not shown) did not kill any of the *B*. *abortus* strains at the tested concentrations. Thus, CCL20 is more potent than β-defensins in terms of killing *B*. *abortus*.

## Discussion

Antimicrobial peptides are considered an important component of the host defense at mucosal surfaces, including the lung. Beta-defensins produced by the respiratory epithelial cells and alveolar macrophages are secreted into the airway surface fluid, where they increase their levels during lung infectious and inflammatory processes. Besides their direct antimicrobial function, β-defensins have multiple roles as mediators of inflammation [47, 48, 60]. Similarly, CCL20 has been shown to be produced by lung epithelial cells [22, 30, 31] and to be induced in the lungs by some infections [30, 31]. Different studies suggest that pulmonary CCL20 could not only exert chemoattractant activities on lymphocytes and dendritic cells [61] but could also exert direct antimicrobial effects [22]. Despite the known roles of β-defensins and CCL20 in the pulmonary defense against infections, and the ability of *Brucella* spp. to infect hosts through inhalation, nothing is known about the expression of these molecules in *Brucella*-infected lung epithelial cells and their antimicrobial activity against this pathogen.

Here we show that *B*. *abortus* induces the expression of CCL20 on human alveolar (A549) or bronchial (Calu-6) epithelial cell lines, and also in primary alveolar epithelial cells obtained from human lungs. These findings agree with similar results obtained for airway epithelial cells infected with other respiratory pathogens [30, 62, 63] or stimulated with microbial antigens [64, 65]. CCL20 is also produced by monocytes/macrophages in response to infections or microbial antigens [54, 66]. This may be relevant during pulmonary infections given the presence of resident alveolar macrophages and the recruitment of monocytes by inflammatory chemokines. In particular, previous studies have shown that *Brucella*-infected airway epithelial cells produce MCP-1, a chemoattractant for monocytes [45]. In the present study we found that the monocytic THP-1 cell line as well as primary monocytes and monocyte-derived macrophages respond to *B*. *abortus* infection with an enhanced production of CCL20. Moreover, the same response was observed when human lung tissue explants were infected with *B*. *abortus* ex vivo, strongly suggesting that an enhanced CCL20 secretion may take place in the lungs of infected patients. Collectively, these results suggest that different cell types may respond to a *B*. *abortus* pulmonary infection with the production of CCL20, potentially contributing to the recruitment of lymphocytes and dendritic cells to the infection site. Of note, the CCL20 levels produced by epithelial cells, monocytic/macrophagic cells and lung explants in response to *B*. *abortus* infection fit well with levels reported to be chemoattractant for dendritic cells and lymphocytes [31, 67–69].

Previous studies have shown a varying contribution of MAPK and NF-κB signaling pathways to the CCL20 response to infections or microbial antigens according to the cell type and/or the pathogen considered. In the present study, CCL20 expression was mainly mediated by JNK1/2 and NF-κB in both Calu-6 and THP-1 *Brucella*-infected cells, with a partial contribution of p38 and ERK1/2 in Calu-6 cells. A previous study demonstrated the involvement of p38 signaling in the CCL20 response of bronchial epithelial cells to *Chamydophila pneumoniae* [62], and other study revealed that both p38 and NF-κB are involved in the CCL20 response of murine lung epithelial cells to *Burkholderia pseudomallei* [30]. These studies, however, did not test the potential involvement of ERK1/2 and JNK1/2 pathways. In line with our findings, all the four signaling pathways have been found to be involved in CCL20 production by gingival fibroblasts in response to *E*. *coli* LPS [55].

The induction of CCL20 secretion by *B*. *abortus* seems to be independent of bacterial viability as it was also markedly induced by heat-killed *B*. *abortus* (HKBA) in A549, Calu-6 and THP-1 cells. These results agree with previous studies showing the induction of different cytokines and chemokines by HKBA in different cell types [50, 56]. Those studies further demonstrated that the responses induced by HKBA were mediated by *B*. *abortus* lipoproteins but not by its LPS, and that they depended on TLR2 signaling. The results of the present study fully agree with such reports as we found that CCL20 secretion is elicited by L-Omp19 (a model *B*. *abortus* lipoprotein) but is not elicited by *B*. *abortus* LPS. Also in agreement with previous studies, CCL20 secretion was TLR2-dependent. The inability of *B*. *abortus* LPS to induce CCL20 secretion by epithelial and monocytic cells agrees with the reported weak proinflammatory activity of this molecule, which has been mainly attributed to the unusual composition of its lipid A [50, 70–72].

In contrast to the results obtained for CCL20, no induction of hBD2 at either the mRNA level or protein level was detected in human lung epithelial cells or monocytes infected with *B*. *abortus*, although hBD2 mRNA expression increased significantly in response to the proinflammatory cytokine IL-1β added as positive control. These results contrast with those found for infections of lung epithelial cells by other pathogens such as *Mycobacterium tuberculosis*, *Klebsiella pneumoniae*, *Pseudomonas aeruginosa* or *Aspergillus fumigatus* [27, 28, 34, 73]. The reasons for the lack of hBD2 induction in A549 cells upon *B*. *abortus* infection are intriguing as A549 was one of the cell lines used in all these previous studies and mRNA induction was detected in every case. Interestingly, however, in most cases hBD2 induction had particular requirements. The mRNA expression of this defensin was induced by a non-capsular mutant of *K*. *pneumoniae* but not by its capsular counterpart, and in the case of *P*. *aeruginosa* only the mucoid phenotype had an inducing effect. In the case of *A*. *fumigatus*, hBD2 expression was higher in cells exposed to swollen conidia, compared to resting conidia or hyphal fragments. Therefore, it seems that the induction of hBD2 in A549 cells and other lung epithelial cells is highly dependent on the composition of the outer membrane of pathogens. *Brucella* LPS is known to have an atypical composition, in particular regarding lipid A backbone and acylation, probably responsible for its low proinflammatory activity [50, 70–72, 74]. Further studies will be needed to determine whether the particular composition of *B*. *abortus* LPS may be responsible for the lack of hBD2 response of lung epithelial cells to this pathogen.

To our best knowledge there are no studies comparing in parallel the production of hBD2 and CCL20 by lung epithelial cells in response to microbes or microbial antigens. In colonic epithelial cells (Caco-2) both molecules were induced (at the mRNA level) by wild type enteropathogenic *E*. *coli*, but only CCL20 was induced by a mutant lacking flagellin [75]. This suggests that, at least in this model, the induction pathways leading to CCL20 expression differed from those involved in hBD2 expression.

While lung epithelial cells did not produce hBD2 in response to *B*. *abortus* infection, it must be kept in mind that in the context of pulmonary infections interactions between these cells and resident or recruited phagocytes may amplify the innate immune response to pathogens. To model this type of interactions we stimulated lung epithelial cells with conditioned medium from *B*. *abortus*-infected monocytes (CMB). Notably, we found that hBD2 expression was significantly induced in A549 cells by CMB, and a similar inducing effect was observed on CCL20 secretion. Experiments using blocking agents revealed that IL-1β, but not TNF-α, was involved in the induction of hBD2 and CCL20 secretion by CMB. These findings resemble those previously reported by us for chemokine responses of A549 cells and human bronchial epithelial cells to *B*. *abortus* infection [45]. In that previous study, lung epithelial cells had a null or poor proinflammatory response to direct *B*. *abortus* infection but exhibited a marked increase of chemokine production (IL-8 and MCP-1) upon stimulation with CMB. IL-1β was shown to mediate these inductions in A549 cells and TNF-α in bronchial epithelial cells. In line with these previous findings, in the present study IL-1β was shown to mediate CCL20 and hBD2 induction by CMB in A549 cells. These results also agree with previous reports showing that IL-1β induces CCL20 and hBD2 secretion by pulmonary epithelial cells [34, 57, 58]. Whereas in these studies TNF-α also induced the production of these molecules, such effect was not noted in the present study. It must be noted, however, that in two previous studies [34, 57] stimulation was performed with recombinant TNF-α and not with conditioned medium. In the other study, in which A549 cells were stimulated with conditioned medium from LPS-stimulated monocytes, IL-1β was a more potent inducer of hBD2 than TNF-α [58].

Beside their immunomodulatory functions, both hBD2 and CCL20 have been shown to have direct antimicrobial activity against certain Gram-positive and Gram-negative bacteria [23, 34]. However, the antimicrobial activity of these molecules against *B*. *abortus* has never been tested. In the present study we found that CCL20 killed *B*. *abortus in vitro* with a lethal dose (LD) 50 higher than 50 μg/ml, which was much higher than the LD50 dose against *E*. *coli* (1.5 μg/ml). The LD50 dose for *B*. *abortus* was also higher than those reported for several bacteria, including *Pseudomonas aeruginosa*, *Moraxella catarrhalis*, *Enterococcus faecium*, *Staphylococcus aureus* or *Streptococcus pyogenes*, which ranged from 0.2 to 10 μg/ml [23]. Therefore, *B*. *abortus* seems to be comparatively resistant to the antimicrobial action of CCL20. Moreover, the LD50 dose of CCL20 for *B*. *abortus* was much higher than the levels of this chemokine produced by lung epithelial cells infected with *B*. *abortus* or stimulated with CMB. Overall, these results suggest that whereas CCL20 may have chemotactic and immunomodulatory roles during pulmonary *B*. *abortus* infections, it would not have an antimicrobial role against this pathogen.

As lung epithelial cells stimulated with CMB produced hBD2, it was of interest to test a potential antimicrobial action of this defensin against *B*. *abortus*. While the LD50 for *P*. *aeruginosa* and *E*. *coli* have been reported to be around 5 μg/ml [34], *B*. *abortus* resisted hBD2 (and hBD3) doses of up to 50 μg/ml. As mentioned, there are no previous studies on the antimicrobial activity of β-defensins against *Brucella* microorganisms. However, it has been shown that *Brucella* is resistant to antimicrobial peptides from other families, such as polymixn B, melittin (honey bee), magainin (frog skin) and cecropin (pig) [76]. The results of the present study allow expanding the list of antimicrobial peptides that lack bactericidal activity against *B*. *abortus*.

The resistance of smooth strains of *Brucella* to these antimicrobial peptides has been attributed in part to the O polysaccharide in the LPS of these bacteria, as smooth strains (expressing the polysaccharide) are more resistant to cationic peptides (polymyxin B, melittin, cecropin and others) than rough strains (devoid of the polysaccharide) [77]. In the present study, however, a *B*. *abortus* mutant devoid of O polysaccharide (RB51) was no more sensitive than its smooth counterpart to hBD2 concentrations up to 50 μg/ml. In contrast, the lack of O polysaccharide seemed to affect the sensitivity of *B*. *abortus* to CCL20 as the LD50 against this mutant was of 8.7 ug/ml, which is significantly lower than the LD50 against wild type *B*. *abortus*.

Collectively, the results of the present study show that human lung epithelial cells secrete CCL20 and hBD2 in response to *B*. *abortus* and/or to cytokines produced by infected monocytes. Whereas these molecules are produced at concentrations that do not seem to exert antimicrobial activity against this pathogen, they could be involved in immunomodulatory functions during pulmonary *Brucella* infections, such as the recruitment and activation of immune cells.
